# PCR Primer Design for 16S rRNAs for Experimental Horizontal Gene Transfer Test in *Escherichia coli*

**DOI:** 10.3389/fbioe.2017.00014

**Published:** 2017-02-28

**Authors:** Kentaro Miyazaki, Mitsuharu Sato, Miyuki Tsukuda

**Affiliations:** ^1^Department of Life Science and Biotechnology, Bioproduction Research Institute, National Institute of Advanced Industrial Science and Technology (AIST), Tsukuba, Ibaraki, Japan; ^2^Department of Computational Biology and Medical Sciences, Graduate School of Frontier Sciences, The University of Tokyo, Kashiwa, Chiba, Japan

**Keywords:** bacterial phylogeny, 16S rRNA, ribosome, horizontal gene transfer, molecular clock, functional complementation, metagenome, central pseudoknot

## Abstract

We recently demonstrated that the *Escherichia coli* ribosome is robust enough to accommodate foreign 16S rRNAs from diverse gamma- and betaproteobacteria bacteria (Kitahara et al., 2012). Therein, we used the common universal primers Bac8f and UN1541r to obtain a nearly full-length gene. However, we noticed that these primers overlap variable sites at 19[A/C] and 1527[U/C] in Bac8f and UN1541r, respectively, and thus, the amplicon could contain mutations. This is problematic, particularly for the former site, because the 19th nucleotide pairs with the 916th nucleotide, which is a part of the “central pseudoknot” and is critical for function. Therefore, we mutationally investigated the role of the base pair using several 16S rRNAs from gamma- and betaproteobacteria. We found that both the native base pairs (gammaproteobacterial 19A–916U and betaproteobacterial 19C–916G) and the non-native 19A–916G pair retained function, whereas the non-native 19C–916U was defective 16S rRNAs. We next designed a new primer set, Bac1f and UN1542r, so that they do not overlap the potential mismatch sites. 16S rRNA amplicons obtained from the environmental metagenome using the new primer set were dominated by proteobacterial species (~85%). Subsequent functional screening identified various 16S rRNAs from proteobacteria, all of which contained native 19A–916U or 19C–916G base pairs. The primers developed in this study are thus advantageous for functional characterization of foreign 16S rRNA in *E. coli* with no artifacts.

## Introduction

The bacterial ribosome consists of 3 rRNA molecules and 54 proteins and plays a crucial role in translating mRNA-encoded information into proteins. Because of the structural complexity of the ribosome (Schuwirth et al., 2005), it is believed that each ribosomal component coevolves to maintain function (Jain et al., 1999). In particular, because the 16S and 23S rRNAs form the structural core of the ribosome (Schuwirth et al., 2005), they are believed to be least likely to experience horizontal gene transfer between species (Jain et al., 1999). On the basis of the species-specific nature of rRNA and their omnipresence in all bacteria, the rRNA genes, especially those for 16S rRNA, have long been used as an “ultimate chronometer” (Woese, 1987) for phylogenetic classification of bacterial species (Lane et al., 1985; Woese, 1987).

Despite the apparent species-specific nature of 16S rRNAs, we recently found that the *Escherichia coli* ribosome is able to accommodate foreign 16S rRNA (Kitahara et al., 2012). Namely, using *E. coli* Δ7, a null mutant of the *rrn* (ribosomal RNA) operon, as a host strain, we have shown that various 16S rRNA genes, including those from a different phylogenetic class (i.e., betaproteobacteria), were able to complement growth. The lowest identity of functional 16S rRNA gene to that of *E. coli* was as low as 80%, implying that hundreds of simultaneous nucleotide changes are permitted in the maintenance of ribosome function. The basis for this high mutability is the conservation of the RNA secondary structures, which is consistent with a previous finding that 16S rRNA is typically recognized by ribosomal proteins via salt bridges between phosphate oxygen atoms of the RNA backbone, but nucleotide bases are not strictly discriminated (Brodersen et al., 2002). Furthermore, insertion/deletion is allowed in some RNA helices (e.g., h6, 10, and 17) that are not involved in protein binding. Understanding the sequence and structural variations of 16S rRNA that are accommodated in the *E. coli* ribosome should be helpful for our understanding of the evolution of rRNA and the sequence–structure–function relationships of the ribosome.

**Figure 1:**
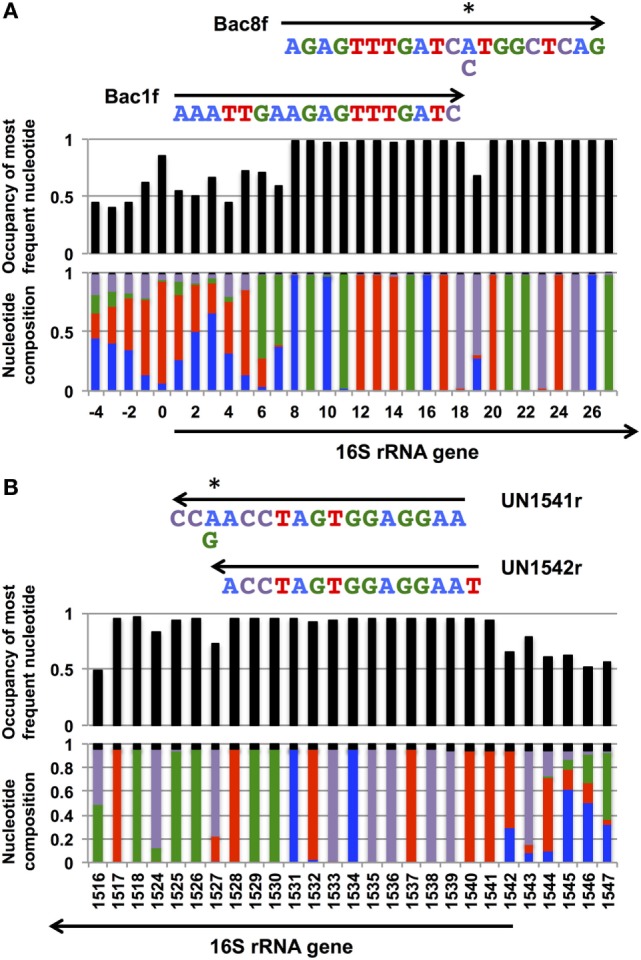
**Coverage rates and nucleotide composition at the (A) 5′- and (B) and 3′-ends of the bacterial 16S rRNA gene**. The annealing regions for Bac1f, Bac8f, and UN1541r are shown by arrows with the sequences. Wobble nucleotides are marked by asterisks. Colors: blue, A; red, T; green, G; purple, C; black, gap.

**Figure 2:**
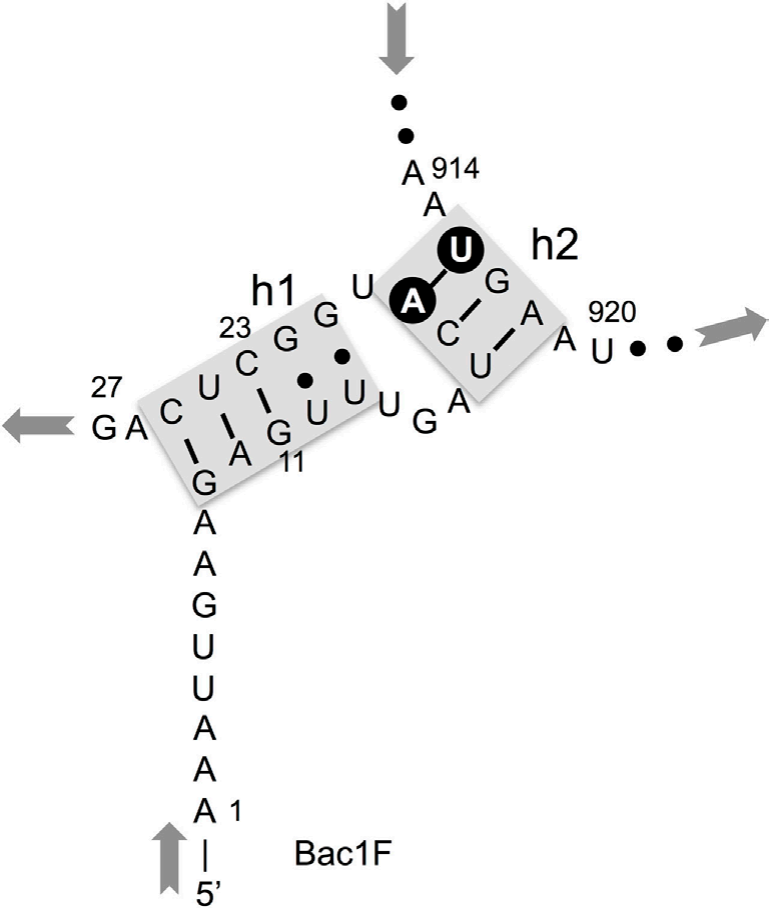
**Central pseudoknot structure in *Escherichia coli* 16S rRNA**. Nucleotide numbers 19 and 916 are labeled in white letters in a black circle.

In our previous study, to PCR amplify foreign 16S rRNA genes, we used Bac8f(A) or Bac8f(C) [the most commonly used “Bac8f” (Eden et al., 1991)] for the forward primer and UN1541r(U) or UN1541r(C) for the reverse primer (Figure 1; oligonucleotide sequences summarized in Table 1) (Kitahara et al., 2012), which allowed amplification of a nearly full-length gene. These primers can cover the majority of bacterial 16S rRNA genes and thus are commonly used for phylogenetic and/or community analysis (Lane et al., 1985; Weisburg et al., 1991; Amann et al., 1995). However, we noticed that the amplicons obtained using the primer set contained mutations at certain frequencies, which could affect the functionality of *in vivo-*reconstituted mutant ribosomes. In *E. coli* 16S rRNA, nucleotides 17–19 pair with nucleotides 916–918 to form a short helix (h2) (Figure 2). The helix is involved in the formation of the “central pseudoknot,” whose structure is highly conserved in both prokaryotes and eukaryotes. This unique structure is essential for translational initiation and is highly susceptible to point mutations (Brink et al., 1993; Dammel and Noller, 1993; Poot et al., 1998). Despite this structural conservation, however, the 19th nucleotide varies depending on the species, 19A or 19C, which pairs with 916U or 916G, respectively (Figure 2). Thus, if Bac8f(A) or Bac8f(C) is used as a primer, there is a possibility of generating a mismatch between the 19th and 916th nucleotides. Similarly, the 1,527th position is also variable (C or U) and can generate a mismatch in the amplicons (Figure 1B), although this site may not be involved in function. Thus, in our specific system for functional investigation of 16S rRNAs, it is essential to develop a new primer set to avoid the introduction of artificial mutations. In addition, we need to take the RNA processing issue into consideration. For proper processing of a precursor transcript into mature rRNAs (16S, 23S, and 5S rRNAs), the processing sites (i.e., RNase cleavage sites) need to be similar to the *E. coli* sequences (Gutgsell and Jain, 2012).

**Table 1:** **PCR primers used for amplification of 16S rRNA genes**.

Primer	Sequence	Reference
Bac1f	5′-AAATTGAAGAGTTTGATC-3′	This study
Bac8f(A)^a^	5′-AGAGTTTGATCATGGCTCAG-3′	Kitahara and Miyazaki (2011); Kitahara et al. (2012); Weisburg et al. (1991)
Bac8f(C)^a^	5′-AGAGTTTGATCCTGGCTCAG-3′	Kitahara and Miyazaki (2011); Kitahara et al. (2012); Weisburg et al. (1991)
UN1542r	5′-TAAGGAGGTGATCCA-3′	This study
UN1541r(U)^a^	5′-AAGGAGGTGATCCAACC-3′	Kitahara and Miyazaki (2011); Kitahara et al. (2012)
UN1541r(C)^a^	5′-AAGGAGGTGATCCAGCC-3′	Kitahara and Miyazaki (2011); Kitahara et al. (2012); Weisburg et al. (1991)
Bac1r	5′-GATCAAACTCTTCAATTTAAAAGTTTGACGCTCAAAG-3′	This study
Bac8r(A)	5′-CTGAGCCATGATCAAACTCTTC-3′	Kitahara and Miyazaki (2011); Kitahara et al. (2012)
Bac8r(C)	5′-CTGAGCCAGGATCAAACTCTTC-3′	This study
UN1542f	5′-TGGATCACCTCCTTACCTTAAAGAAGCGT-3′	This study

*^a^Underlined nucleotides are the sites that can generate a mismatch*.

Taking these points into consideration, we first evaluated the effects of mismatches between the 19th and 916th nucleotides. In addition to the native 19A–916U and 19C–916G pairs, the non-native 19A–916G pair retained function, whereas the non-native 19C–916U was detrimental. Second, we designed new primers, Bac1f and UN1542r, which encompassed nucleotide positions 1–18 for Bac1f and 1542–1528 for UN1542r, so that they did not overlap the potential mismatch sites. These primers were used for PCR amplification of 16S rRNA genes and the resultant library was functionally screened. DNA sequencing of the 16S rRNA genes in the functional clones confirmed the absence of unwanted mismatches in the functional genes.

## Materials and Methods

### Reagents

KOD-Plus-Neo and KOD FX-Neo DNA polymerases were purchased from Toyobo (Osaka, Japan). Trimethoprim (Tmp), ampicillin (Amp), kanamycin (Km), and sucrose (Suc) were purchased from Wako Pure Chemicals (Tokyo, Japan). Zeocin™ (Zeo) and the In-Fusion Cloning Kit were purchased from Invitrogen (Carlsbad, CA, USA). Lennox LB medium [1% (w/v) tryptone, 0.5% (w/v) yeast extract, 0.5% (w/v) NaCl] was purchased from Merck (Tokyo, Japan). The Extrap Soil DNA Kit Plus ver.2 was purchased from J-Bio21 (Tsukuba, Japan). FastDNA Kit was purchased from BIO101 (La Jolla, CA, USA). Oligonucleotide primers (Table 1) were purchased from Sigma (Hokkaido, Japan).

### Bacterial Strains and Culture Conditions

The following bacterial strains were purchased from the Biological Resource Center (NBRC), National Institute of Technology and Evaluation, Japan: *Serratia ficaria* (NBRC 102596), *Caldimonas manganoxidans* (NBRC 16448), *Hydrogenophaga flava* (NBRC 102514), *Hydrogenophilus thermoluteolus* (NBRC 14978), *Oxalicibacterium horti* (NBRC 13594), *Oligella urethralis* (NBRC 14589), and *Ralstonia pickettii* (NBRC 102503). *Burkholderia sacchari* was a laboratory stock. Competent *E. coli* JM109 cells were purchased from RBC Bioscience (Taipei, Taiwan). Antibiotics were added when necessary at the following concentrations: Tmp, 10 μg/ml; Amp, 100 μg/ml; Km, 25 μg/ml; and Zeo, 50 μg/ml. Agar (1.5% [w/v]) was added to solidify the medium. Suc was added at 5% (w/v) for counterselection purposes when necessary.

### Genomic DNA Purification, PCR Amplification of 16S rRNA Genes, and Library Construction

Genomic DNA of bacterial isolates was purified using the FastDNA Kit. The *Nitrosomonas europaea* was a gift from Dr. Naohiro Noda (AIST, Japan). Environmental metagenomic DNA (soils, fermented products, and seawater) was purified using the Extrap Soil DNA Kit Plus ver. 2. The 16S rRNA genes were amplified by PCR using a set of primers, Bac1f, Bac8f(A), or Bac8f(C) and UN1542r. The reaction mixture contained 100 ng of template DNA (bacterial genome or environmental metagenome), 1× PCR buffer, 0.4 mM each of dNTPs, 0.25 μM each of primers and 1 U of KOD FX-Neo DNA polymerase in a total volume of 50 μl. The mixture was heated at 94°C for 2 min and subjected to 30 rounds of thermal cycling at 98°C for 10 s, 48°C for 30 s for Bac1f or 57°C for 30 s for Bac8f(A) and Bac8f(C), and 68°C for 1.5 min and final incubation at 68°C for 5 min. The amplicon was separated by agarose gel (0.8% [w/v]) electrophoresis; a single band was excised from the gel, purified and dissolved in 30 μl of water.

An expression vector for the 16S rRNA gene was modified from pRB103 (Kitahara and Suzuki, 2009; Kitahara et al., 2012) by deleting the genes for tRNA, 23S rRNA, and 5S rRNA, replacing the antibiotic selection marker from Zeo to Tmp and replication origin from pSC101 to p15A. The resultant plasmid was named pMS205aTp1 (map illustrated in Figure S1A in Supplementary Material). The entire vector (without the 16S rRNA gene) was PCR-amplified using the primer set Bac1R, Bac8r(A), or Bac8r(C) and UN1542f. The PCR mixture contained 1× PCR buffer, 0.2 mM each of dNTPs, 1.5 mM MgSO_4_, 0.25 μM each of primers, 10 ng of pMS205aTp1, and 1 U of KOD-Neo-DNA polymerase in a total volume of 50 μl. The mixture was heated at 94°C for 2 min and subjected to 25 cycles at 94°C for 10 s, 60°C for 30 s, and 68°C for 2.5 min, followed by a final incubation at 68°C for 5 min. The products were treated with *Dpn*I (10 U, 37°C, 6 h), gel-purified and dissolved in 30 μl of water.

The 16S rRNA gene (*ca*., 200 ng) and the linearized pMS205aTp1 (*ca*., 200 ng) fragments were combined and ligated using the In-Fusion Cloning Kit in a total volume of 10 μl. After incubation at 50°C for 1 h, the reaction products (2 μl) were introduced into competent *E. coli* JM109 cells (100 μl) and grown on LB/Tmp agar plates at 37°C overnight. Some of the colonies were randomly picked and used for sequence determination for phylogenetic analysis. Rest of the colonies were combined and plasmids were extracted to yield a library.

### Functional Screening of 16S rRNA Genes

*Escherichia coli* MY201 *rna^−^* is a derivative of *E. coli* Δ7 *rna^−^* (Kitahara and Suzuki, 2009), which contains the growth rescue plasmid pMY201 (modified from pRB101 by substituting the pSC101 ori to p15A ori, Figure S1B in Supplementary Material) and pML103Δ (expression plasmid for 23S rRNA, 5S rRNA, and tRNAs, created by deleting the 16S rRNA gene from pRB103, map illustrated in Figure S1C in Supplementary Material). Competent MY201 cells were transformed with a pMS205aTp1 library and grown on LB/Km/Zeo/Tmp agar plates at 37°C for overnight. Colonies were collected, mixed in 1 ml of LB broth, vigorously vortexed, appropriately diluted, and spread over LB/Km/Zeo/Tmp/Suc agar plates. Some of the colonies were randomly picked and used for sequence determination for phylogenetic analysis. Rest of the colonies grown on the plates were collected and used for further studies.

### Growth Assay

Mutant *E. coli* strains were grown in 1 ml of LB/Km/Zeo/Tmp/Suc broth in a 96 deep-well plate. The plate was incubated at 37°C with vigorous agitation (1,200 rpm) in an MBR-024 microplate shaker (Taitec, Saitama, Japan). After 14 h, 1 μl of the culture was transferred to a fresh LB/Km/Zeo/Tmp/Suc broth (1 ml) in 96-well plate and grown at various temperatures (30, 37, or 42°C) with vigorous agitation (1,200 rpm). After 14 h, 200 μl of the culture was transferred to a 96-well plate and OD_600_ was measured.

### DNA Sequencing and BLAST Search

DNA sequencing was carried out using the Sanger method with an Applied Biosystems (Foster City, CA, USA) automatic DNA sequencer (ABI PRISM 3130xl Genetic Analyzer) and an Applied Biosystems BigDye (ver. 3.1) kit. Blast search (Altschul et al., 1990) was carried out using the NCBI nucleotide database “16S rRNA sequences (Bacteria and Archaea)” with the program selection optimized for “Highly similar sequences (megablast).”

### Dataset and Sequence Alignment of 16S rRNA Genes

All 16S rRNA gene sequences (plus 50 additional nucleotides at the 5′ and 3′ ends) were retrieved from the genomic sequences in the NCBI database (as of August 2014) (Table 2). A total of 9,624 genes were identified in 2,476 genomes of 23 phyla. Multiple sequence alignment of these genes was performed using the MAFFT v7 program (Katoh and Standley, 2013).

**Table 2:** **List of 16S rRNA genes retrieved from the NCBI database**.^a^

Phylum or group	Number of genomes	Number of 16S rRNA genes
Actinobacteria	268	833
Aquificae	13	27
Bacteroidetes–Chlorobi group	99	287
Caldiserica	1	1
Chlamydiae–Verrucomicrobia group	112	200
Chloroflexi	20	34
Chrysiogenetes	1	3
Deferribacteres	4	7
Cyanobacteria	75	168
Dictyoglomi	2	4
Deinococcus–Thermus	22	51
Elusimicrobia	1	1
Fibrobacteres–Acidobacteria group	7	12
Firmicutes	532	3,304
Fusobacteria	9	38
Nitrospirae	4	7
Planctomycetes	7	18
Proteobacteria	1,144	4,369
Spirochaetes	58	106
Synergistetes	4	11
Tenericutes	74	111
Thermodesulfobacteria	2	3
Thermotogae	17	29
Total	2,476	9,624

*^a^All 16S rRNA gene sequences were extracted from all available genomic sequences in NCBI database on August 1, 2014*.

### Nucleotide Sequence Accession Numbers

The nucleotide sequences for 16S rRNA gene have been deposited in GenBank/EMBL/DDBJ under the accession numbers LC213146–LC213207, LC213207–LC213252, and LC213253–LC213296.

## Results and Discussion

### Primer Design

Nucleotide composition around the 5′- and 3′-end regions of all bacterial 16S rRNA genes (Table 2) is shown in Figure 1. For the 5′-end, the sequence surrounding the 19th nucleotide, particularly from the 8th to 27th, is highly conserved (Figure 1A), which corresponds to the Bac8f primer-binding site. The Bac8f primer covers 97% of bacterial 16S rRNA sequences (Figure 1A), confirming the appropriateness of the primer for phylogenetic/community analysis (Lane et al., 1985; Amann et al., 1995). However, due to the presence of a potential mismatch site at the 19th nucleotide position, this is not appropriate for our specific purpose (i.e., functional analysis), and thus, we designed a new primer Bac1f, which encompasses the 1st to 18th nucleotides. Although the very beginning of the sequence (from first to seventh nucleotides) is highly variable among all bacteria (Figure 1A), the region is critical for RNA processing (Gutgsell and Jain, 2012), so we strictly followed the *E. coli* sequence for this site.

We next checked the coverage rate of the Bac1f primer for each phylum (Figure S2 in Supplementary Material). As described above, the 5′ end of the 16S rRNA sequence varies among bacteria (Figure 1A). Nevertheless, Bac1f showed relatively high specificity to some bacterial 16S rRNAs that included Bacteroidetes–Chlorobi (Figure S2B in Supplementary Material), Chlamydiae-Verrucomicrobia (Figure S2C in Supplementary Material), and Proteobacteria (Figure S2G in Supplementary Material). In our previous study (Kitahara et al., 2012), no functional 16S rRNAs were obtained from phyla other than proteobacteria. Thus, in practice, although the Bac1f primer has some bias to specific phylogenetic groups, this bias is advantageous to enriching libraries with a potentially functional fraction and to reducing background.

Figure S3 in Supplementary Material summarizes the coverage rate of the Bac1f primer for each class of proteobacteria. Overall, there is a slight preference for alpha-, beta-, and gamma-classes of proteobacteria, and the delta-epsilon class has a larger number of potential mismatches.

### Effects of Non-Natural Base Pairing between the 19th and 916th Nucleotides on Ribosomal Activity

To investigate how non-natural base pairing between the 19th and 916th nucleotides affects ribosomal activity, we used 16S rRNA genes from the following bacteria: gammaproteobacterial *E. coli* (Eco) and *S. ficaria* (Sfi) and betaproteobacterial *B. sacchari* (Bsa), *C. manganoxidans* (Cma), *H. flava* (Hfl), *H. thermoluteolus* (Hth), *N. europaea* (Neu), *O. horti* (Oho), *O. urethralis* (Our), and *R. pickettii* (Rpi).

PCR amplification was carried out using three types of forward primers: Bac1f, Bac8f(A), or Bac8f(C); UN1542r was used as a common reverse primer. A specifically amplified fragment was then cloned back into pMS205aTp1. After confirming the sequence of the entire 16S rRNA gene, the resultant plasmid was transferred into *E. coli* MY201 *rna^−^* (Δ7 strain). After *sacB-*based counterselection to eliminate rescue plasmids expressing *E. coli* 16S rRNA, all the clones were successfully obtained at 37°C, implying that mutation in the 19th–916th base pair is more or less permissive under this condition.

**Figure 3:**
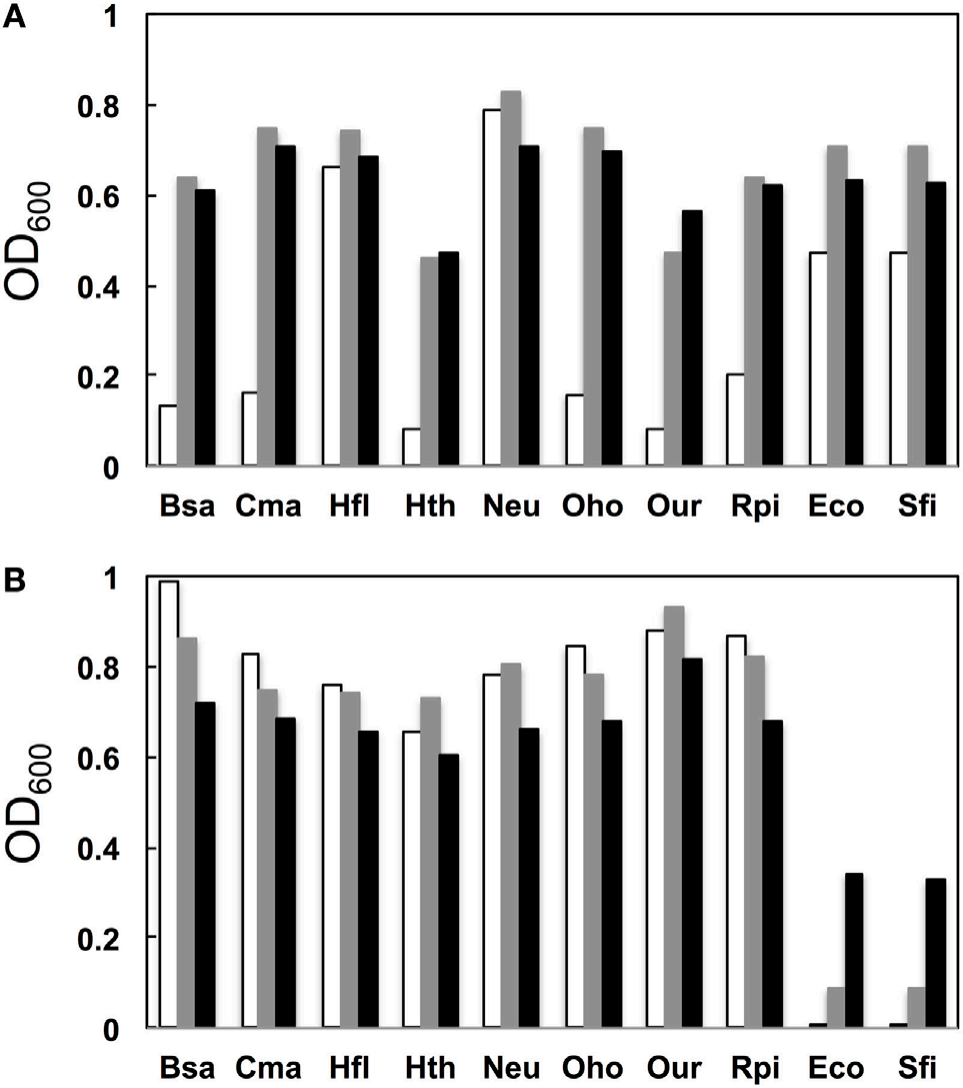
**Growth profile**. **(A)** Native base pairs between nucleotides 19 and 916 [19C–916G for betaproteobacterial *Burkholderia sacchari* (Bsa), *Caldimonas manganoxidans* (Cma), *Hydrogenophaga flava* (Hfl), *Hydrogenophilus thermoluteolus* (Hth), *Nitrosomonas europaea* (Neu), *Oxalicibacterium horti* (Oho), *Oligella urethralis* (Our), and *Ralstonia pickettii* (Rpi) and 19A–916U for gammaproteobacterial *Escherichia coli* (Eco) and *Serratia ficaria* (Sfi)] and **(B)** non-native base pairs between nucleotides 19 and 916 (19A–916G for betaproteobacterial Bsa, Cma, Hfl, Hth, Neu, Oho, Our, and Rpi and 19C–916U for gammaproteobacterial Eco and Sfi). *y* axis represents OD_600_ after cultivation at various temperatures (30°C, open bars; 37°C, shaded bars; 42°C, solid bars) for 14 h.

We next examined the growth properties of each mutant at various temperatures (30°C, 37°C, and 42°C). Figure 3A illustrates OD_600_ after growth for 14 h. In general, for the native base pairs (19A–916U for gammaproteobacterial and 19C–916G for betaproteobacterial 16S rRNAs) all clones had higher OD_600_ at 37°C than at 30°C. For the clones carrying non-native pairs (Figure 3B, 19C–916U for gammaproteobacterial and 19A–916G for betaproteobacterial 16S rRNAs), no growth perturbation was observed for betaproteobacterial clones and they appeared to gain a broadened temperature optimum; final OD_600_ shifted upward at 30°C. In contrast, gammaproteobacterial clones (Eco and Sfi) showed greatly reduced OD_600_ values at all temperatures. Virtually no growth was observed at 30°C, indicating the appearance of a cold-sensitive (or heat-tolerant) phenotype.

Poot et al. (1998) have analyzed the role of h2 through mutagenesis. Using *E. coli* 16S rRNA as a template, they introduced a point mutation to alter the native 19A–916U base pair to 19A–916G, 19C–916G, and 19C–916U. They used the mutant ribosome in an *in vitro* translational assay (at 42°C) and found that the former two mutants retained nearly full activity (>80%), whereas 19C–916U had much reduced (30%) activity. Although the assay systems are different, the general conclusion of both their study and ours is that 19C–916U is defective.

### Metagenomic Screening for Functional 16S rRNA Genes in *E. coli*

**Figure 4:**
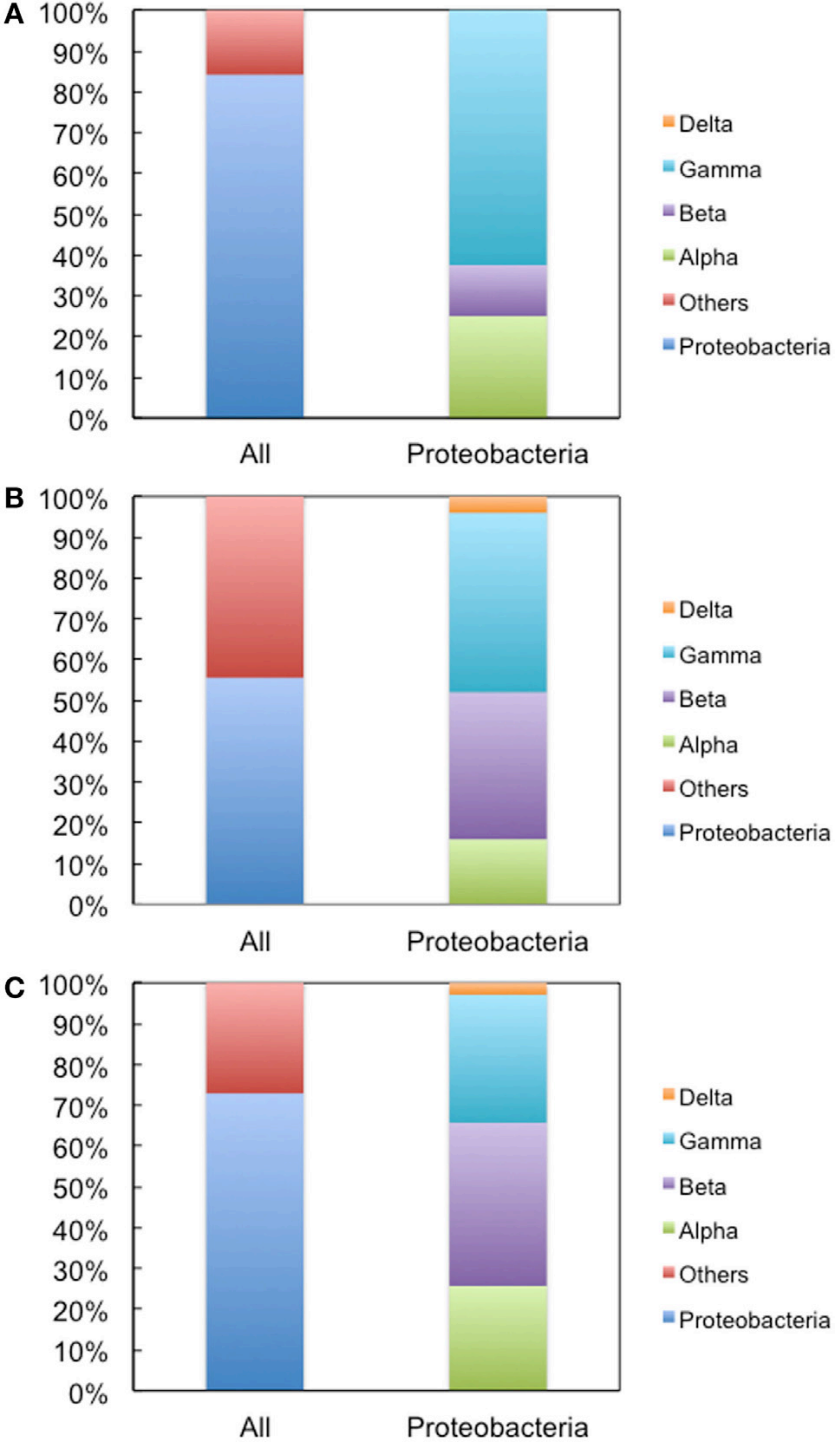
**Phylogenetic analysis of 16S rRNA genes cloned in pMS205aTp1 (before functional selection) amplicons**. The forward primers used were **(A)** Bac1f, **(B)** Bac8f(A), and **(C)** Bac8f(C). “All” is the classification based on phylum and “Proteobacteria” is the class composition in the proteobacterial phylum.

We next used environmental metagenomes as a source for 16S rRNA genes. For all primer sets, specific amplification was obtained (Figure S4 in Supplementary Material). To investigate sequence diversity, the cloned genes were phylogenetically characterized. As shown in Figure 4, when Bac1f was used as a forward primer, the gene was dominated by proteobacterial 16S rRNAs [~84% (32/38)], but was much less for those obtained using the Bac8f(A) and Bac8f(C) primers [~56% (25/45) and ~73% (35/48), respectively].

The genes were then subjected to functional screening. Functional 16S rRNA genes were collected, and their microbial origins and the base pair patterns between the 19th and 916th nucleotides were investigated. As shown in Table 3, when Bac1f was used, 85% (52/61) of functional 16S rRNAs were from gammaproteobacteria and the rest were from betaproteobacteria. Phylogenetic tree of these sequences is illustrated in Figure S5A in Supplementary Material. Base pair patterns were 78% 19A–916U (48/61) and 22% (13/61) 19C–916G. It is noteworthy that no artificially shuffled base pairs (A–G and C–U) were observed, implying that the newly designed primers did not introduce non-native base pairs and are adequate for functional studies. There are some mismatches between proteobacterial 16S rRNA and Bac1f sequences (Figure S3 in Supplementary Material), but in practice, we succeeded in retrieving various functional genes, suggesting that the mismatches at the 5′-end can affect annealing efficiency, but still remain effective for amplification. The lack of any alphaproteobacterial 16S rRNAs in our functional 16S rRNA collection may be due to functional incompatibility in *E. coli*.

**Table 3:** **Sequence analysis of functional 16S rRNA genes in *Escherichia coli Δ*7 retrieved from the metagenome**.

	Bac1f	Bac8f(A)	Bac8f(C)
**Predicted microbial origins**			
Gammaproteobacteria	52	39	13
Betaproteobacteria	9	6	29
Deltaproteobacteria	0	0	2
Total	61	45	44
**Base pairs between the 19th and 916th nucleotides**			
A–G	0	21	0
A–U	48	23	1
C–G	13	1	42
C–U	0	0	1
Total	61	45	44

When Bac8f(A) was used as a forward primer, 87% (39/45) of functional 16S rRNAs were of gammaproteobacteria and the rest (13%; 6/45) were of betaproteobacteria. Phylogenetic tree of these sequences is illustrated in Figure S5B in Supplementary Material. When the base pair patterns were investigated, non-canonical base pairs were frequently observed. Approximately half (21/45) of the sequences contained the 19A–916G base pair, which may have resulted from the mis-annealing of the primer to template 16S rRNA genes containing 19C and 916G. Because this artificial base pair is permissive (or even encouraged) under normal growth conditions (Figure 3B), it is reasonable to find these species; simple PCR conditioning may not easily remove these mis-annealed products.

When Bac8f(C) was used as a forward primer, 66% (29/44) of functional 16S rRNAs were of betaproteobacteria, 30% (13/44) were of gammaproteobacteria, and 5% (2/44) were of deltaproteobacteria. Phylogenetic tree of these sequences is illustrated in Figure S5C in Supplementary Material. In this library, non-natural base pair patterns were rarely observed. Most were of the 19C–916G base pair and the non-native 19C–916U base pair was found in one clone (closest relative was gammaproteobacterial *Rahnella aquatilis*, NR_074921). This low-level occurrence of the 19C–916U base pair agreed with the detrimental (yet still non-lethal) effect of the pair on cell growth (Figure 3B).

In conclusion, we developed a new primer set, Bac1f and UN1542r, for the functional study of 16S rRNAs in *E. coli*. The effective utilization of the primers was demonstrated by retrieval of a range of functional 16S rRNAs from the proteobacterial lineage, all of which contained a native base pair between the 19th and 916th nucleotides.

## Author Contributions

KM, MS, and MT designed the study, conducted the data analysis, and wrote the manuscript.

## Conflict of Interest Statement

The authors declare that the research was conducted in the absence of any commercial or financial relationships that could be construed as a potential conflict of interest.
