# Supplementary material for: PCR Primer Design for 16S rRNAs for Experimental Horizontal Gene Transfer Test in Escherichia coli
Source: Front Bioeng Biotechnol. 2017 Feb 28;5:14. doi: 10.3389/fbioe.2017.00014 (PMC5329695; doi:10.3389/fbioe.2017.00014)
Supplement: Supplementary file 1 [file Presentation_1.PDF]

Supplemental materials for

PCR primer design for 16S rRNAs for experimental horizontal gene transfer test in *Escherichia coli*

Kentaro Miyazaki, Mitsuharu Sato and Miyuki Tsukuda

FIG. S1. Plasmid map of (A) pMS205aTp1, (B) pMY201 and (C) pML103Δ.

The map was drawn by using a plasmid editor software (ApE v2.0.47).

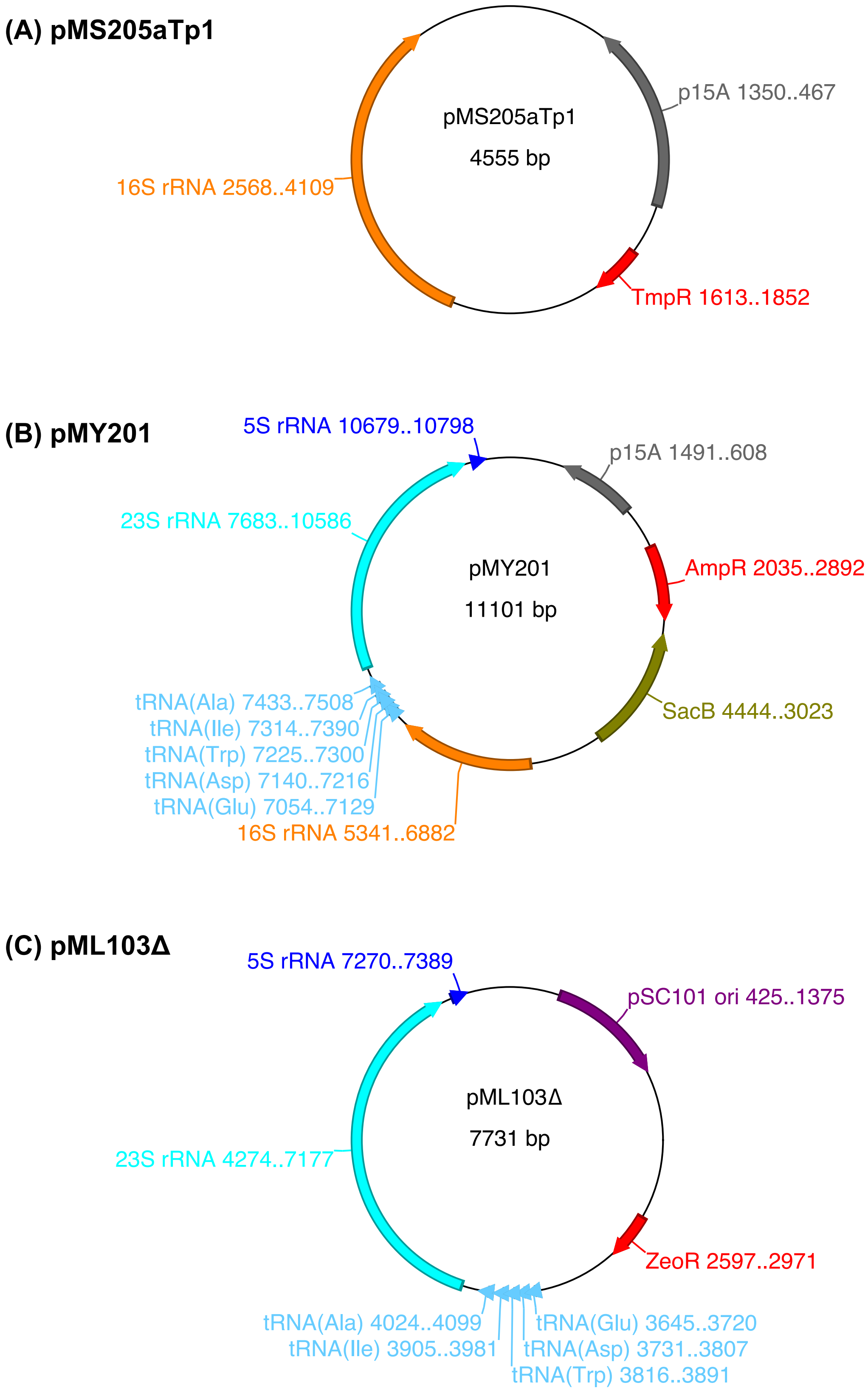

FIG. S2. Nucleotide sequence and base composition at the 5'-end Bac1f priming site of 16S rRNA of various bacterial phyla.

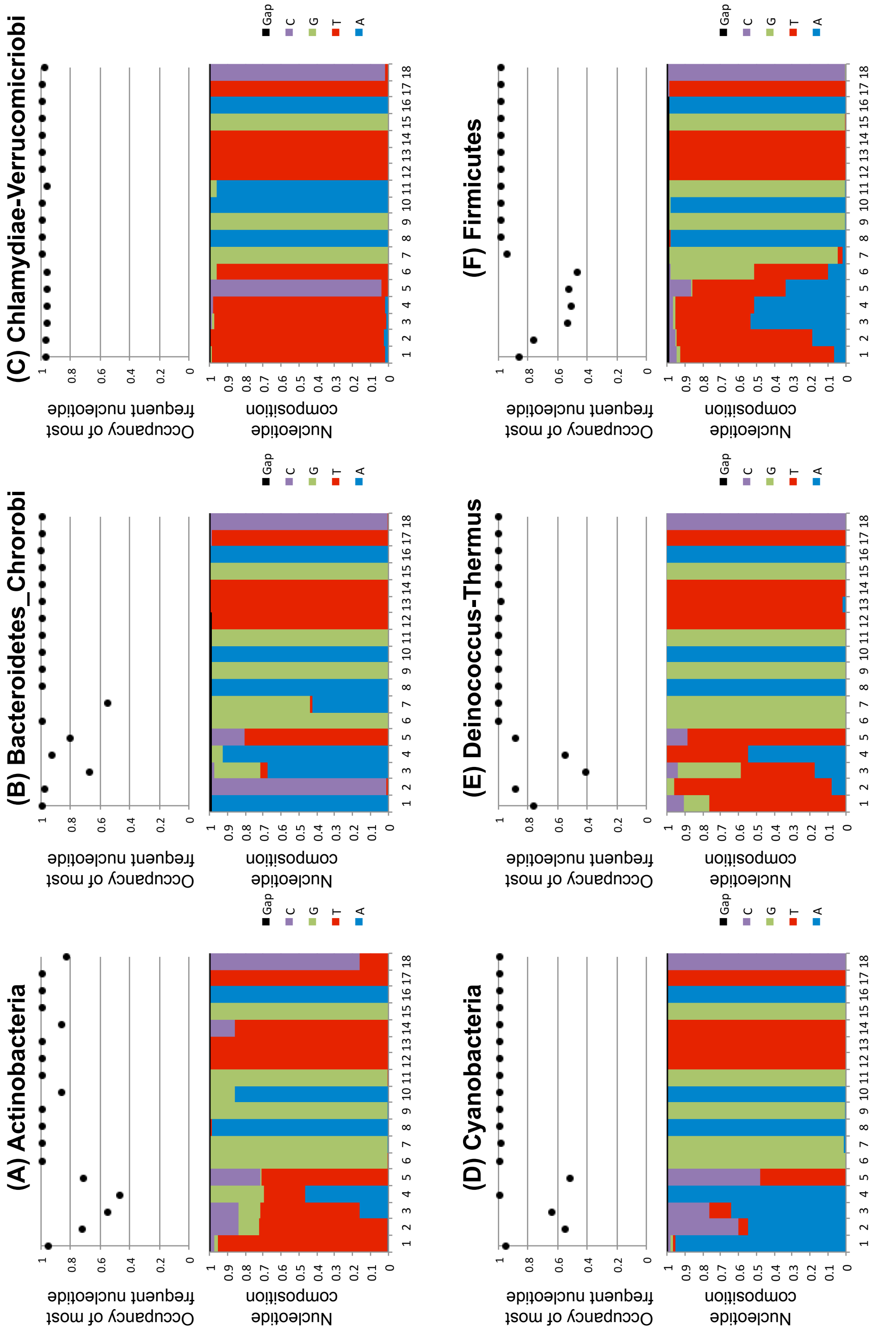

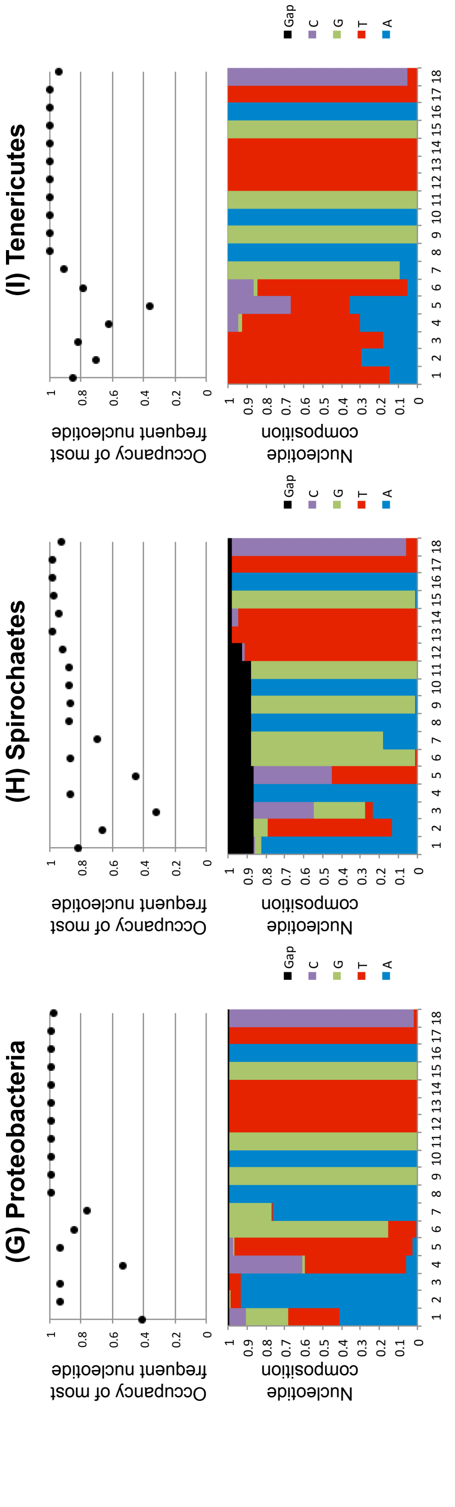

(G) Proteobacteria
(H) Spirochaetes
(I) Tenericutes
Occupancy of most frequent nucleotide
Nucleotide composition
Gap
C
G
T
A

FIG. S3. Nucleotide sequence and base composition at the 5'-end Bac1f priming site of the proteobacterial 16S rRNA gene. (A) All proteobacteria; (B) alphaproteobacteria; (C) betaproteoobacteria; (D) gammaproteobacteria; and (E) delta- and epsilonproteobacteria.

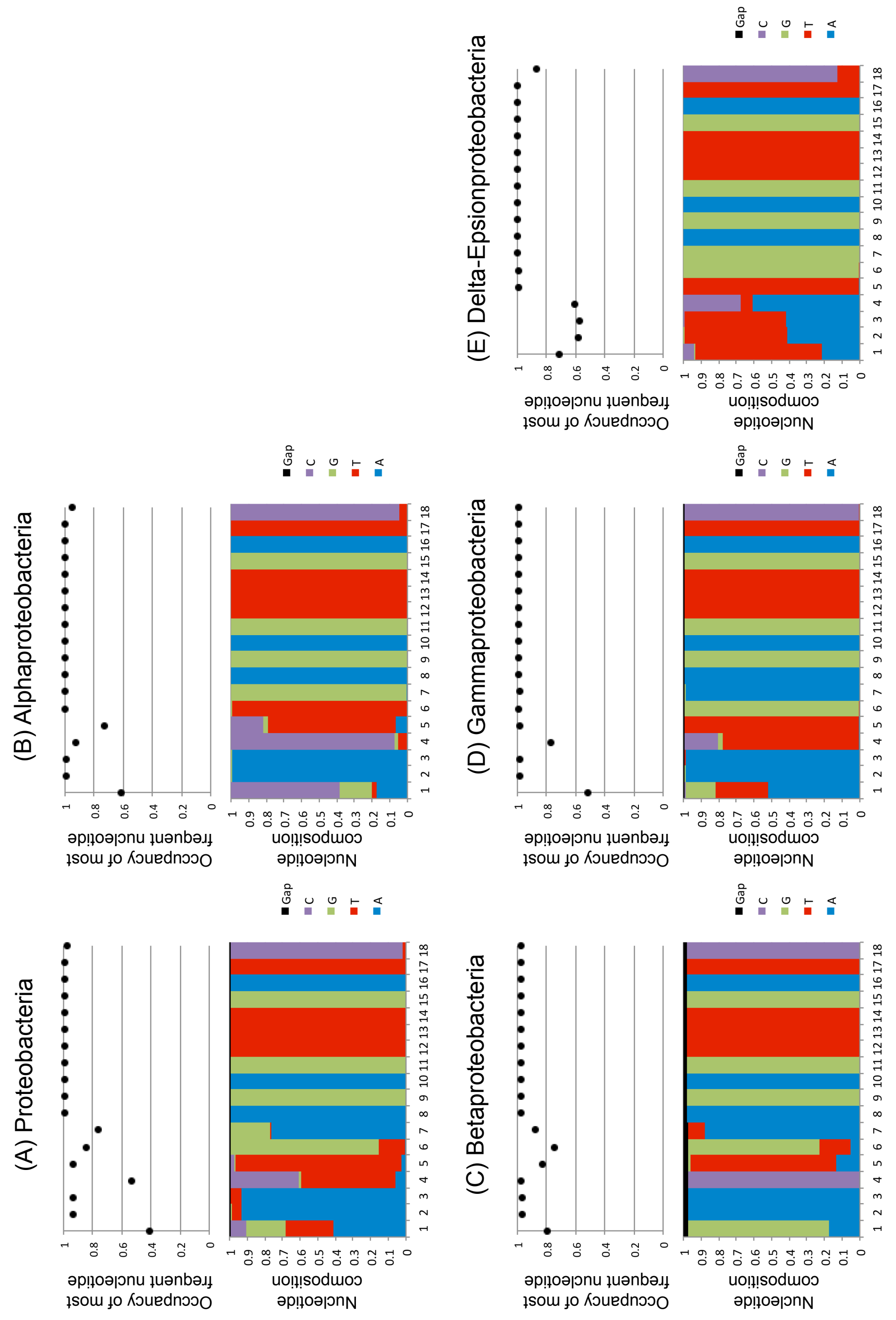

FIG. S4. Agarose gel electrophoresis of PCR products. PCR was carried out using different forward primers: Bac1f (lane 1), Bac8f(A) (lane 2) and Bac8f(C) (lane 3) in conjunction with UN1542r as a common reverse primer and the environmental metagenome as a template. PCR conditions are described in the Materials and Methods section.

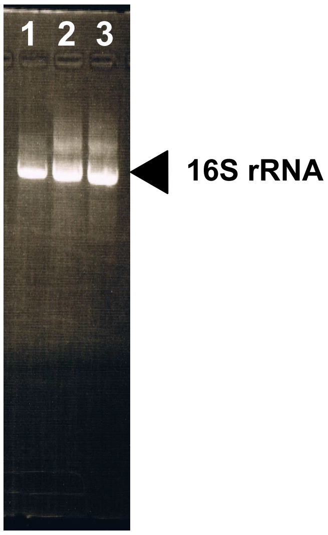

FIG. S5. Phylogenetic tree of functional 16S rRNA genes. Isolated from the libraries that were created by using (A) Bac1f, (B) Bac8f(A), and (C) Bac8f(C) as a forward primer and UN1542r as a reverse primer.

(A)

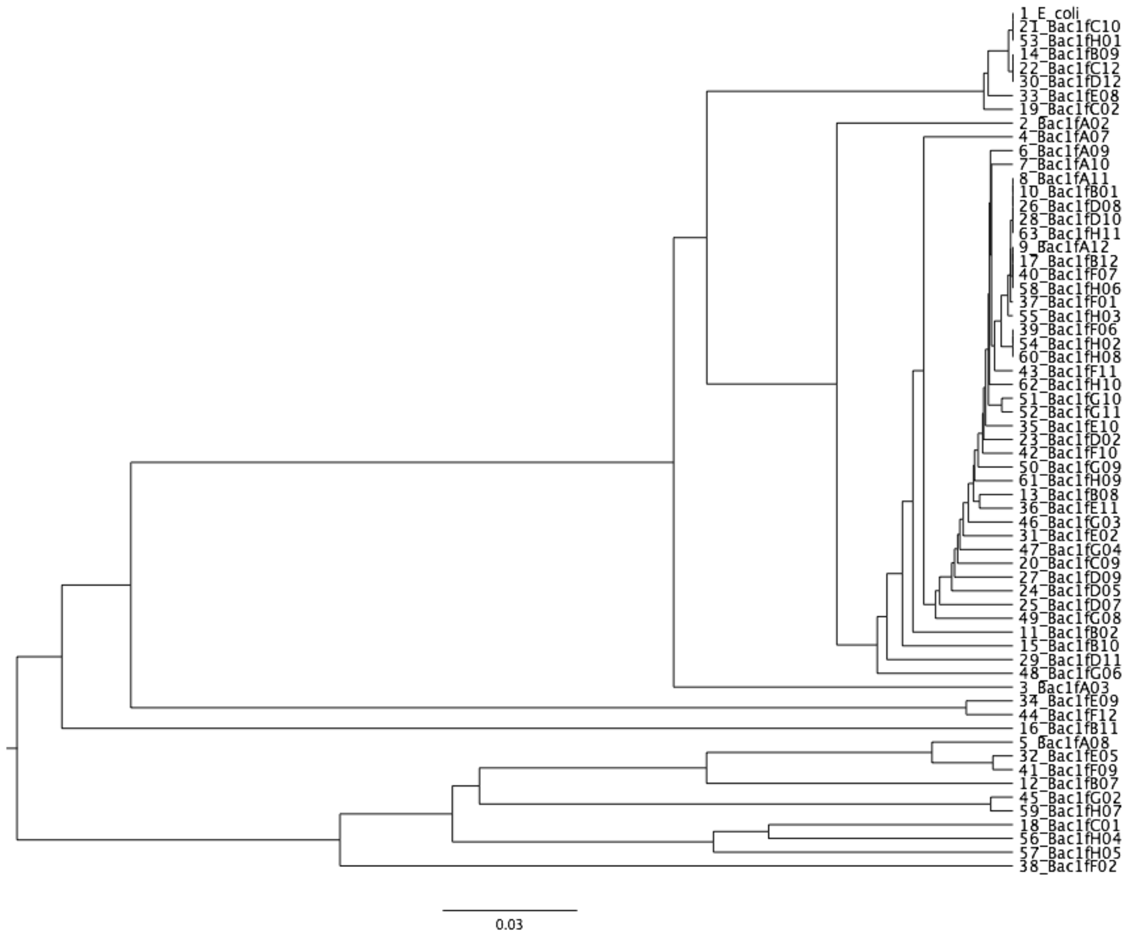

(B)

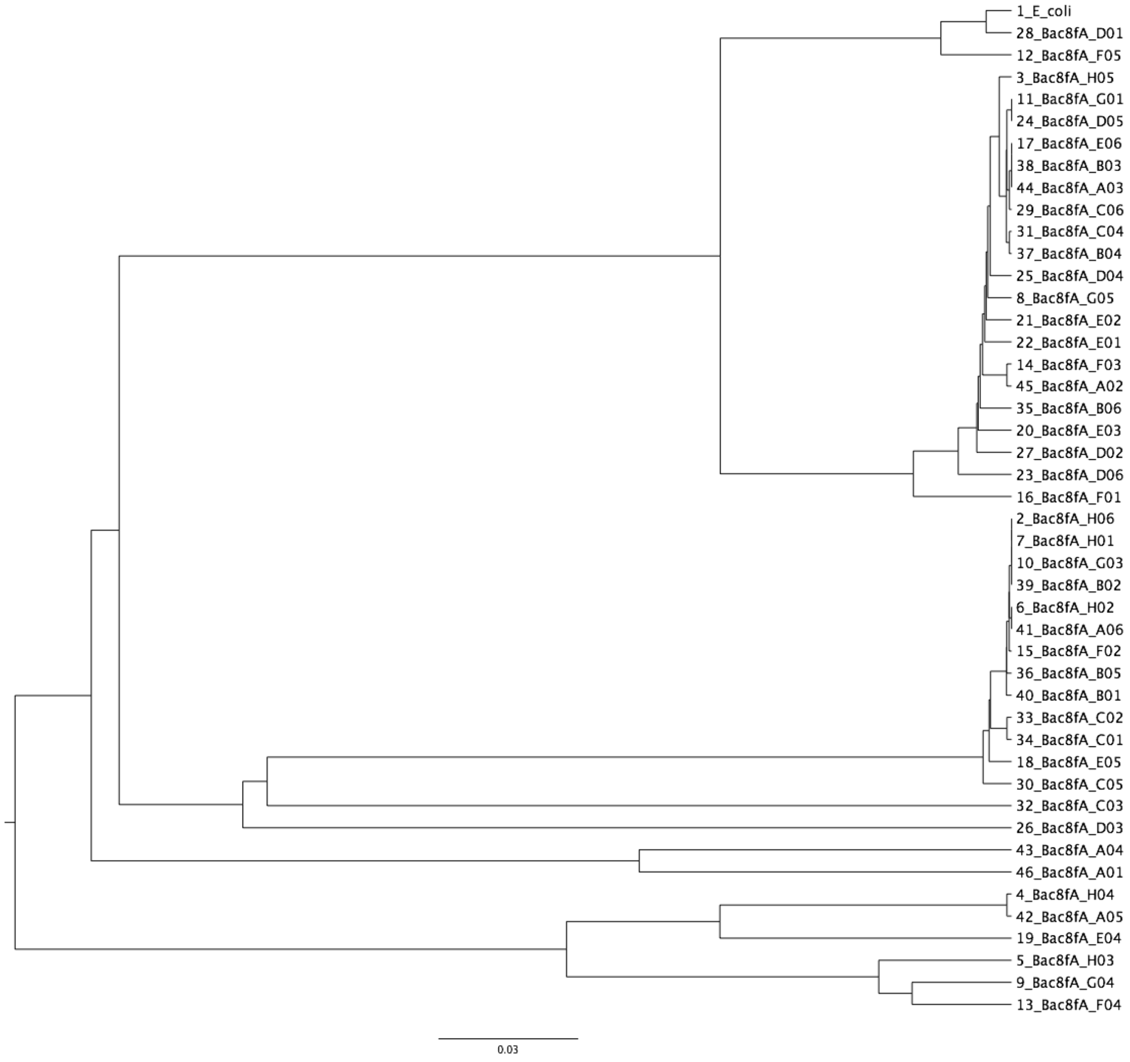
1_E_coli
28_Bac8fA_D01
12_Bac8fA_F05
3_Bac8fA_H05
11_Bac8fA_G01
24_Bac8fA_D05
17_Bac8fA_E06
38_Bac8fA_B03
44_Bac8fA_A03
29_Bac8fA_C06
31_Bac8fA_C04
37_Bac8fA_B04
25_Bac8fA_D04
8_Bac8fA_G05
21_Bac8fA_E02
22_Bac8fA_E01
14_Bac8fA_F03
45_Bac8fA_A02
35_Bac8fA_B06
20_Bac8fA_E03
27_Bac8fA_D02
23_Bac8fA_D06
16_Bac8fA_F01
2_Bac8fA_H06
7_Bac8fA_H01
10_Bac8fA_G03
39_Bac8fA_B02
6_Bac8fA_H02
41_Bac8fA_A06
15_Bac8fA_F02
36_Bac8fA_B05
40_Bac8fA_B01
33_Bac8fA_C02
34_Bac8fA_C01
18_Bac8fA_E05
30_Bac8fA_C05
32_Bac8fA_C03
26_Bac8fA_D03
43_Bac8fA_A04
46_Bac8fA_A01
4_Bac8fA_H04
42_Bac8fA_A05
19_Bac8fA_E04
5_Bac8fA_H03
9_Bac8fA_G04
13_Bac8fA_F04
0.03

(C)

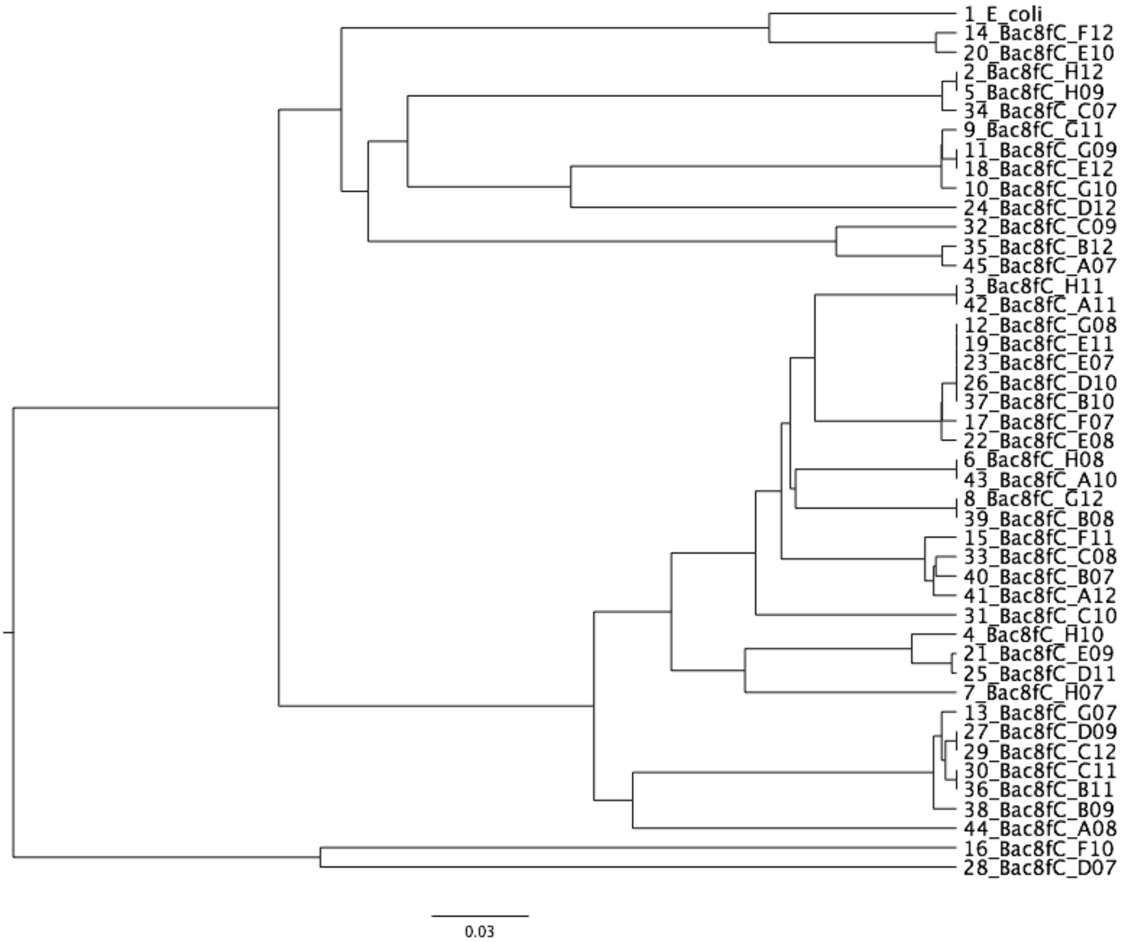
1_E_coli
14_Bac8fC_F12
20_Bac8fC_E10
2_Bac8fC_H12
5_Bac8fC_H09
34_Bac8fC_C07
9_Bac8fC_G11
11_Bac8fC_G09
18_Bac8fC_E12
10_Bac8fC_G10
24_Bac8fC_D12
32_Bac8fC_C09
35_Bac8fC_B12
45_Bac8fC_A07
3_Bac8fC_H11
42_Bac8fC_A11
12_Bac8fC_G08
19_Bac8fC_E11
23_Bac8fC_E07
26_Bac8fC_D10
37_Bac8fC_B10
17_Bac8fC_F07
22_Bac8fC_E08
6_Bac8fC_H08
43_Bac8fC_A10
8_Bac8fC_G12
39_Bac8fC_B08
15_Bac8fC_F11
33_Bac8fC_C08
40_Bac8fC_B07
41_Bac8fC_A12
31_Bac8fC_C10
4_Bac8fC_H10
21_Bac8fC_E09
25_Bac8fC_D11
7_Bac8fC_H07
13_Bac8fC_G07
27_Bac8fC_D09
29_Bac8fC_C12
30_Bac8fC_C11
36_Bac8fC_B11
38_Bac8fC_B09
44_Bac8fC_A08
16_Bac8fC_F10
28_Bac8fC_D07
0.03
